# Color Doppler ultrasound in high-low risk pregnancies and its relationship to fetal outcomes: a cross-sectional study

**DOI:** 10.3389/fped.2023.1221766

**Published:** 2024-02-20

**Authors:** Snehil Dixit, Nitin Arun Dixit, Anil Rawat, Akanksha Bajpai, Magbool Alelyani, Zia Ul Sabah, Shailendra Raghuwanshi

**Affiliations:** ^1^ Department of Medical Rehabilitation Sciences, College of Applied Medical Sciences, King Khalid University, Abha, Saudi Arabia; ^2^ Department of Radiodiagnosis, King George Medical University, Lucknow, India; ^3^ Department of Radiodiagnosis, Career Institute of Medical Sciences, Lucknow, India; ^4^ Department of Radiological Sciences, College of Applied Medical Science, King Khalid University, Abha, Saudi Arabia; ^5^ Department of Medicine, College of Medicine, King Khalid University, Abha, Saudi Arabia; ^6^ Department of Radiodiagnosis, Himalayan Institute of Medical Sciences, Dehradun, India

**Keywords:** gestational stage, color Doppler, high-risk pregnancies, fetal outcome, low-risk pregnancies, Doppler indices

## Abstract

**Objective:**

To calculate the multivessel color Doppler indices in high-risk and low-risk pregnancies and relate these to fetal outcomes.

**Methods:**

The investigation involved 60 patients who were pregnant. The patients were separated into groups according to assessment of low and high risk. The patients underwent color Doppler ultrasonography to detect the maternal and fetal blood vessels, and the measured Doppler indices were then analyzed for any association with fetal outcomes.

**Results:**

The gestational stages (in weeks) of the participants at the respective times of investigation and delivery were 32.06 ± 2.98 and 36.2 ± 1.78 in the low-risk group and 29.21 ± 1.95 and 29.83 ± 1.86 in the high-risk group. The pulsatility index (PI), resistive index (RI), and systolic/diastolic ratio (SD) decreased with gestation length in the low-risk group, whereas in the high-risk group, these values increased in the uterine and umbilical arteries. With increased gestational stage, MCA-PSV (peak systolic velocity) in the middle cerebral artery (MCA) increased, while PI decreased. Pulsatile and reversal flow of the uterine vein, the vein of Galen, and the umbilical vein were noted in high-risk pregnancies, and these negatively affected the fetal outcome. The fetal venous parameters were more specific and sensitive for predicting an unfavorable fetal outcome than the arterial factors, with a greater negative predictive value.

**Conclusion:**

The results of our study indicate that abnormal Doppler indices of the blood vessels in high-risk pregnant patients will result in adverse clinical outcomes. Therefore, the patients can be monitored and managed accordingly using Doppler ultrasonography.

## Highlights of the study

• The fetal venous parameters were more accurate for predicting a poor outcome for the fetus.
• Negative clinical outcomes are associated with high-risk pregnant individuals having abnormal Doppler blood vessel indices.
• High-risk pregnancies were accompanied by pulsatile and reverse flow of the uterine artery, uterine vein, the vein of Galen, and the umbilical vein.

## Introduction

Pre-eclampsia, abruptio placenta, intrauterine growth restriction (IUGR), and fetal death are prominent consequences of poor or malformed placentation, and all have severe impacts on perinatal mortality and morbidity rates. Evidence suggests that a malfunctioning spiral artery or inadequate trophoblastic invasion cause increases in the flow resistance of the uterine arteries, resulting in an improper placentation (1). In fetuses with intrauterine growth limitation brought on by placental insufficiency, blood flow is redirected from the periphery to the brain (2). This risk factor may be identified with appropriate antenatal surveillance, and therapeutic interventions can be undertaken.

With the introduction of color Doppler ultrasonography, monitoring the maternal and fetal blood vessels and predicting complications has become more convenient. Studies have shown a 38% reduction in perinatal death associated with Doppler ultrasonography-guided clinical management (3). Doppler ultrasound studies aid in calculating fetal acid-base status, uteroplacental circulation of the uterine vessels, and neonatal complications (4–6). Uterine artery Doppler ultrasonography can be used to screen for adverse pregnancy outcomes in the first and second trimesters of pregnancy according to extensive studies conducted over the past 20 years (7–10). In the middle cerebral artery and umbilical artery, Doppler ultrasonography can indicate aberrant indices (MCA) and reversals of the flow through the ductus venosus followed by pulsatile umbilical venous flow (DV), indicators of infant death and cardiac failure (11–15). In addition, abnormal maturation of the fetal adrenal artery can be detected, indicating prolonged fetal hypoxia and IUGR (16).

The maternal venous system experiences a substantial increase in blood volume, a factor that is of huge importance during pregnancy (17, 18). These changes in the venous system contribute to maintaining uteroplacental perfusion and regulating maternal cardiac function. Color Doppler imaging can be used to assess the flow velocity of venous system pulsations of the blood vessels, an indicator of complications in the pregnancy (17, 18). Notably, multivessel maternal monitoring of high-risk pregnancies and fetal Doppler ultrasonography have not yet been carried out. There appears to be a knowledge gap regarding the precise comprehension of changes in uteroplacental and fetal circulation when using these Doppler imaging results to forecast perinatal outcomes and direct an appropriate therapy. Using multivessel color Doppler indices, the current study estimated fetal indices in low- and high-risk pregnancies and to demonstrated a link between these indices and fetal outcomes.

## Materials and methods

The current study was a hospital-based observational study undertaken by the Department of Radiodiagnosis at the Himalayan Institute of Medical Sciences (HIMS), Dehradun, to assess color Doppler indicators in high- and low-risk pregnancies. The ethical clearance number for the study was HIHTU/HIMS/ETHICS/2013/40. After receiving permission from the institution's ethics committee, informed consent was acquired before the commencement of the trial, which lasted one year. The two groups of patients– low-risk and high-risk– totaled 60 individuals.

Siemens ACUSON 300 and Siemens G-50 ultrasound systems with a 3.5/5-MHz curvilinear array were used to perform the vessel grey-scale and color Doppler assessments. Patients in the low-risk group had blood pressure less than 140/90 mmHg, a history of giving birth to a live baby weighing more than the fifth percentile, and no history of a medical condition, pre-eclampsia, prenatal hypertension, stillbirth, or abruption.

Patients with pre-eclampsia, a history of pre-eclampsia, abruption, stillbirth/early neonatal death, chronic hypertension, diabetes or renal illness, maternal age greater than 35 years, previous history of intrauterine death, and patients who delivered a baby weighing less than the fifth percentile in prior pregnancies were included in the high-risk category. Participants with a history of numerous pregnancies and a prenatal diagnosis of chromosomal or structural abnormalities were barred from participating in the research.

After receiving written informed consent, participants were clinically assessed, and a grey scale ultrasonography was performed to determine whether they met the study's inclusion or exclusion criteria. Measurements of the femur length, head circumference, biparietal diameter, and abdomen circumference were made using grey-scale ultrasonography. The estimated fetal weight was determined using the Shepard and Hadlock formulae. The details were written down on a Performa constructed explicitly for the experiment.

**Figure 1:**
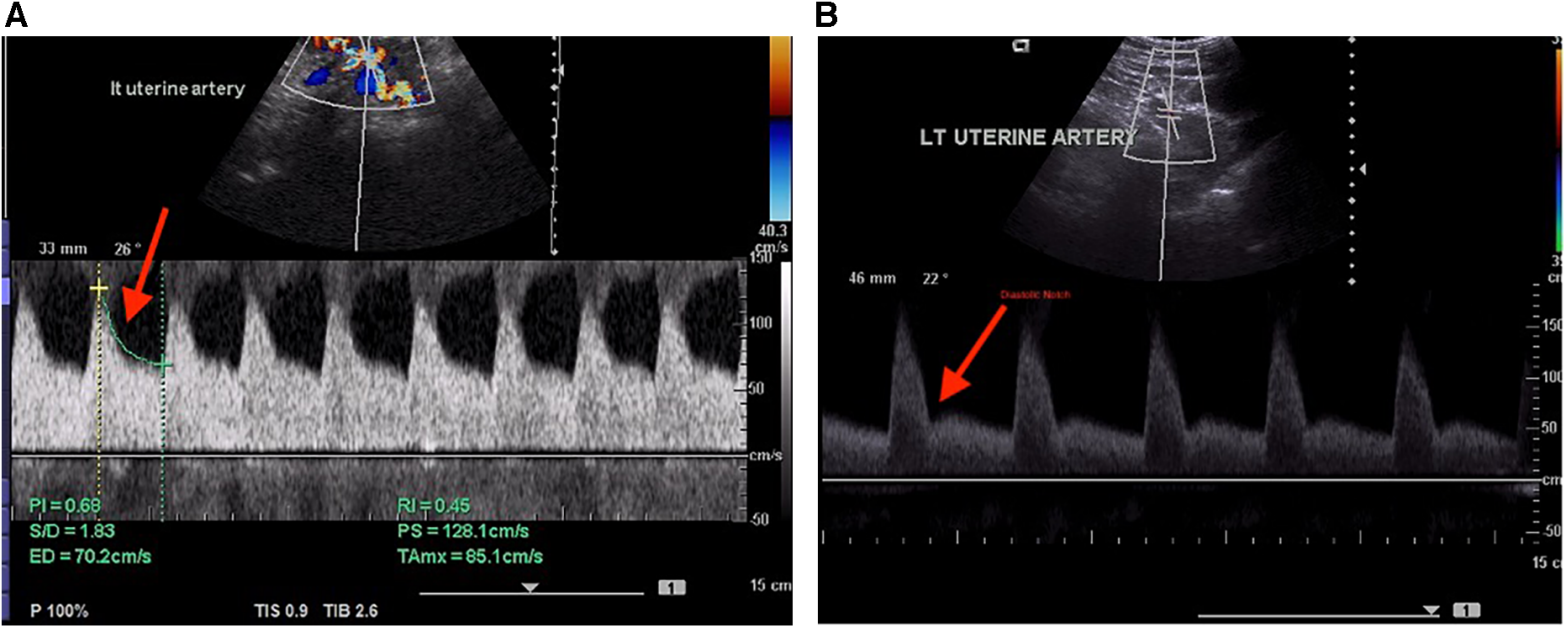
(**A**) Normal Doppler indices in the uterine artery. (**B**) Abnormal uterine artery spectral waveform indicating the presence of a diastolic notch.

The operational definition of an adverse fetal outcome in the present study denoted birth outcomes other than a healthy pregnancy and live birth. The most common such outcomes were small for gestational age, low birth weight (LBW), stillbirth, and premature delivery. The fetal outcome was related to the indices after the vasculature was examined using color Doppler ultrasonography. Waveforms of the uterine arteries from the placental side were recorded in all the examined patients. Diastolic notch, resistive index (RI), pulsatility index (PI), and early diastolic uterine artery notching were all measured. A diastolic notch or PI over the 95th percentile were considered signs of an abnormal Doppler waveform. The uterine veins and uterine arteries as well as their intersection with the iliac vessels were investigated cranially. Uterine vein flow velocity patterns were classified into three categories: Type I was a non-pulsatile, continuous flow; Type II was a pulsatile flow with a “notch” down to the zero line, and Type III, absent flow during a portion of the heart cycle with a pulsatile pattern (Figure 1).

In the fetal proximal region of the MCA, peak systolic velocity (PSV) and PI at the level of the lesser wing of the sphenoid bone were recorded. The PSV and pulse from Galen's vein (GV), transverse sinus (TS), and straight sinus (SS), if any, were noted. The umbilical artery's central, free-floating section was used to measure the umbilical venous blood velocity and blood flow. The S/D ratio, PI, and RI were assessed in the umbilical artery. If any umbilical venous pulsations were observed, they were defined as a diastolic decline in blood velocity greater than 15% of the maximum value at baseline. Using a pulsed Doppler signal, the ductus venosus (DV) was located in the fetal abdomen. Mean velocities were reported for each.

Fetal weight below the 10th percentile was considered small for gestational age (SGA). Metabolic issues, delivery mode, Apgar score, birth weight at five minutes, and newborn intensive care unit (NICU) hospitalization are examples of variables. Analysis of the perinatal outcome in the high-risk group included fetal distress, perinatal and neonatal death, and the requirement for mechanical intubation.

### Data analysis

Version 16 of the Statistical Program for Social Sciences (SPSS) version 20 was used to analyze the data. Chi-square and Fisher's exact tests were used in comparisons of low- and high-risk categories. Analysis of continuous and categorical demographic data was done using the Mann–Whitney *U*-test and Chi-square tests. A *p*-value < 0.05 was considered significant. We calculated the Doppler-based data's PPV, NPV, specificity, and sensitivity.

## Results

The present study was conducted to estimate color Doppler indices and the association with the fetal outcome in high and low-risk pregnant participants. In our study, 33% of patients in the low-risk group belonged to the 20–24 years age group, whereas 53% in the high-risk group belonged to the 30–34 years age group. Sixty percent of the patients in each group were pregnant for the first time (Table 1). During the examination, the gestational ages (in weeks) at examination and delivery were 32.06 ± 2.98 and 36.2 ± 1.78, respectively, in the low-risk group and 29.21 ± 1.95 and 29.83 ± 1.86, respectively, in the high-risk group. In 83.3% of patients, the high-risk group underwent cesarean section, of which 33.3% were emergency, whereas spontaneous delivery (80%) was dominant in the low-risk group.

**Table 1:** Profiles of women in the low- and high-risk groups.

Characteristic	Low risk (*n* = 30)	High risk (*n* = 30)
Age (in years)		
<20	2 (6.66%)	0
20–24	10 (33.33%)	2 (6.66%)
25–29	12 (40%)	11 (36.66%)
30–34	6 (20%)	16 (53.33%)
35 and above	0	1 (3.33%)
Gravida		
Primigravida	18 (60%)	18 (60%)
Multigravida	12 (40%)	12 (40%)

Among the many risk factors, we observed IUGR (73.3%) followed by pre-eclampsia (70%), combined IUGR and pre-eclampsia (43.3%), obesity (20%), history of eclampsia (13.3%), and chronic hypertension (6.6%). None of the patients reported smoking or alcohol consumption. When these risk variables were compared to the fetal outcome, there were significant associations between birth weight, short for gestational age (SGA), neonatal mortality, and APGAR score at five minutes (Table 2).

**Table 2:** Comparison of low-risk (LR) and high-risk (HR) groups for fetal outcome.

Perinatal outcome	Low risk (*n* = 30)	High risk (*n* = 30)	Significance
Apgar score at 5 min < 7	2	22	0.000
SGA	9	27	0.015
Birth weight (g)	2,578.66 ± 444.34	1,624.53 ± 524.70	0.001
Fetal distress	2	6	0.183
NICU admission	1	17	0.001
Neonatal mortality	1	6	0.072
Hyperbilirubinemia	2	6	0.183
Stillbirth	0	2	0.163

Blood vessels were visualized with color Doppler ultrasonography. In our study, we were able to visualize the uterine artery, MCA artery, and umbilical artery in all 60 patients. The adrenal artery was found in 45% of low-risk births and 72% of high-risk pregnancies. A value of less than 5% (2 SD) of the mean was deemed abnormal in the low-risk category.

The mean RI, PI, and S/D ratios of the uterine artery in the low-risk group were 0.43 ± ± 0.05, 0.78 ± 0.20, and 0.79 ± 0.15, respectively, compared to 0.55 ± 0.09, 1.21 ± 0.33, and 2.65 ± 0.53 in the high-risk group. The differences in results were clinically significant (*p* < 0.001). In the high-risk group, abnormalities in uterine artery PI, RI, and S/D were seen in 80%, 70%, and 77% of patients, respectively. There was no significant relationship between uterine artery parameters and perinatal outcome in our study. The indicators decreased with increasing gestation in the low-risk group but increased in the high-risk group.

The type 1 uterine vein waveforms of each patient in the low-risk group were all normal. In the high-risk group, 66% of patients had type 1 waveforms, whereas 20% and 13.3% had type 2 and 3 waveforms, respectively. There was a statistically significant (*p* < 0.001) association between patients with pulsatile uterine vein velocity and newborn birth weight. Neonatal death was always the result of fetal distress, which was present in 60% of the individuals with pulsations.

**Figure 2:**
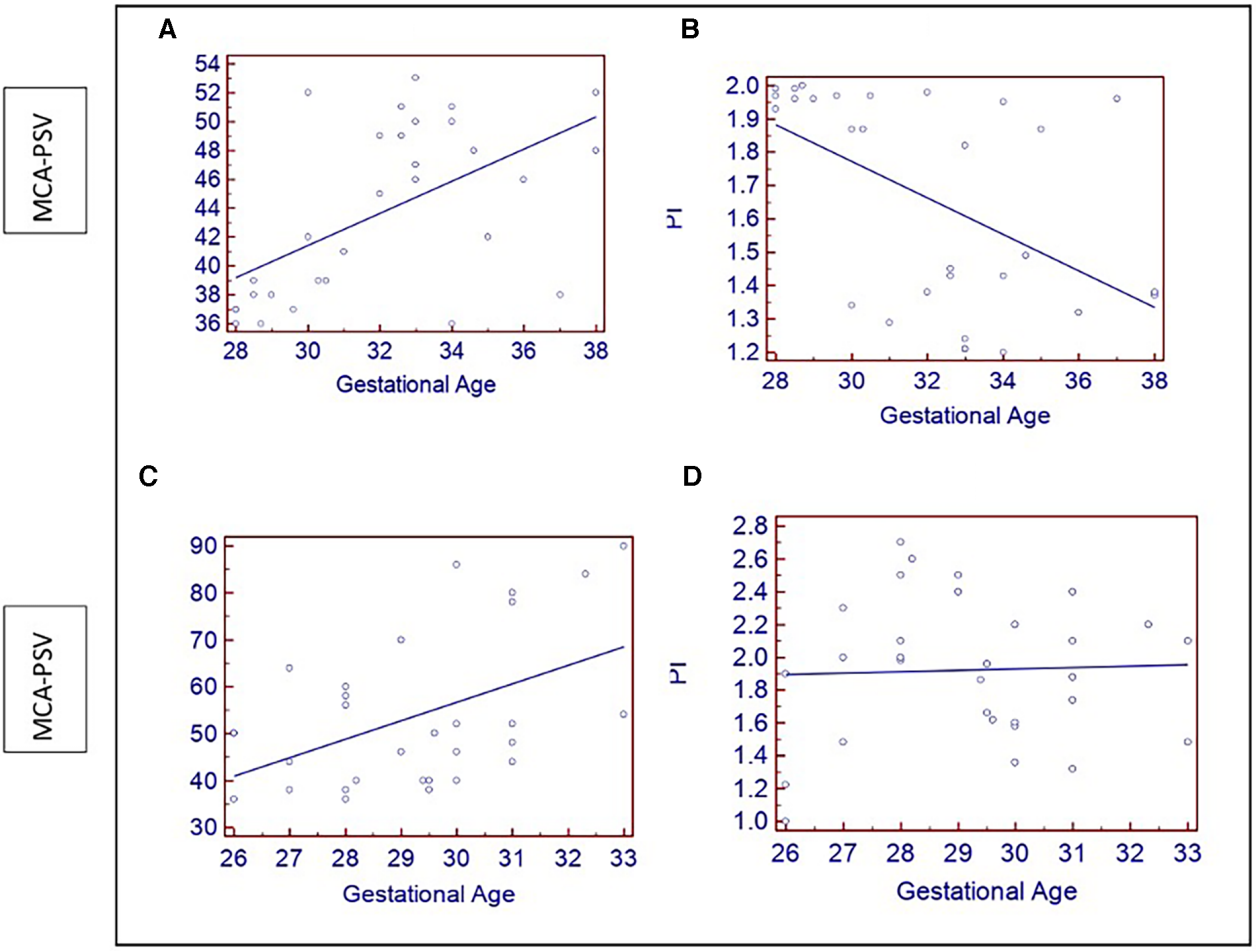
(**A–C**) Show scatter plots of increasing fetal MCA –PSV (cm/s) with gestational age (weeks) in both groups. (**B**) A scatter plot showing the of decrease in PI with gestational age in the low-risk group; (**D**) Shows no changes in the PI of the high-risk group.

In the low-risk and high-risk groups, the MCA artery's mean PSV and PI ratios were 43.73 ± 5.91 and 1.66 ± 0.33, 53.6 ± 16.08, and 1.45 ± 0.35, respectively (*p* < 0.05). Anomalies in the PI and PSV occurred in 40% and 33.3% of patients in the high-risk group, respectively. The aberrant indices were significantly associated with birth weight, admission to the NICU, fetal distress, and newborn death (*p* < 0.05). Both groups showed increases in PSV as gestation progressed. Although the patients in the low-risk group had lower PI levels, the high-risk group did not significantly vary from the low-risk group (Figure 2).

The fetal brain displayed the straight sinus (SS), transverse sinus (TS), and vein of Galen, all showing an increase in velocity with the development of gestation. The pulsations of the vein of Galen were statistically significant (*p* = 0.012) compared to the SS and TS, which were not statistically significant (*p* > 0.05) when the venous pulsations were observed and compared between the groups. The pulsatility of the vein of Galen was significantly correlated with birth weight, NICU hospitalization, and hyperlipidemia fetal distress leading to perinatal death (*p* < 0.05). However, such an association was not observed for the perinatal outcome and pulsatility of the sphenoid and transverse sinus (*p* > 0.05).

In the low-risk group, the mean RI, PI, and S/D ratios of the umbilical artery were 0.60 ± 0.06, 0.92 ± 0.14, and 3.09 0.50, respectively, whereas in the high-risk group, these were 0.70 ± 0.11, 0.92 ± 0.14, and 4.44 ± 1.70, respectively (*p* < 0.001). In the low-risk group, the indices declined as gestation advanced; however, RI and PI did not change significantly despite an increase in the SD ratio in the high-risk group. In the high-risk group, 60%, 46%, and 39% of the individuals had abnormal PI, RI, and S/D results, respectively. There was a significant association between the abnormal indices and the perinatal outcome (Table 3). There was a normal umbilical vein flow pattern among all participants in the low-risk group. Among the high-risk group, 80% of patients had normal flow; 13.3% showed pulsatile flow, and 6% had reverse flow. Abnormal pulsations were significantly associated with neonatal mortality, birth weight, and fetal distress.

**Table 3:** Normal and abnormal umbilical artery indices in the high-risk group.

Perinatal outcome	High risk pregnancy (*n* = 30)								
	N- PI (*n* = 17)	Ab-PI (*n* = 13)	*P*-value	N- RI (*n* = 16)	Abn-RI (*n* = 14)	*P*-value	N- S/d (*n* = 17)	Ab S/D (*n* = 13)	*P*-value
Birth weight (g)	1,803.5 ± 423.6	1,390.4 ± 566.8	0.02	1,838.5 ± 411.8	1,380.2 ± 545.8	0.01	1,803.5 ± 423.6	1,390.46 ± 566.80	0.02
SGA	15	12	0.162	14	13	0.49	15	12	0.93
Apgar score at 5 min < 7	9	13	0.693	9	13	0.86	9	13	0.26
Fetal distress	1	5	0.393	1	5	0.21	1	5	0.07
NICU admission	4	13	0.446	3	14	0.06	4	13	0.001
Hyperbilirubinemia	0	6	0.01	0	6	0.04	0	6	0.11
Stillbirth	0	2	0.11	0	2	0.22	0	2	0.11
Neonatal mortality	1	5	0.07	1	5	0.21	1	5	0.07

The average peak velocity index for veins (PVIV) ratio of the fetal ductus venosus in the high-risk group was 0.70 ± 0.48, compared to 0.49 ± 0.18 in the low-risk group. The comparison was statistically significant (*p* = 0.028). In addition, 23.3% of patients had abnormal PVIV, and this significantly affected the perinatal outcome (*p* = 0.004). The mean PI and RI values of the fetal aorta in the high-risk group were 1.92 ± 0.53 and 0.56 ± 0.20, respectively, compared to 0.56 ± 0.20 and 0.70 ± 0.15 in the low-risk group. No significant differences were observed for RI or PI with the progression of gestation in both groups. In the high-risk group, the mean PI of the adrenal artery was 1.01 ± 0.16 compared to 0.96 ± 0.11 in the low-risk group. Additionally, 27% exhibited an aberrant PI, although this was only statistically associated with the perinatal outcome for patients in the high-risk group.

High sensitivity for predicting SGA was established by virtually all of the Doppler indices. Complete (100%) negative predictive value for pulsatility of the umbilical vein and the vein of Galen; 100% sensitivity was demonstrated by the umbilical artery PI, the umbilical artery S/D ratio, MCA peak velocity, and pulsatility in uterine and umbilical veins (aberrant DV PVIV waveforms are predictive of an abnormal APGAR score in pregnancies). Abnormal uterine artery parameters, the umbilical vein, and the vein of Galen pulsations had 100% positive predictive value and specificity for fetal distress and perinatal and neonatal death (Table 4).

**Table 4:** Efficacy of Doppler parameters in predicting Perinatal and neonatal mortality.

Variables	Sensitivity	Specificity	PPV	NPV
Uterine artery PI	33	100	100	27
Uterine artery RI	28	100	100	37
Uterine artery S/D	36	100	100	36
Umbilical artery PI	53	94	87	72
Umbilical artery RI	50	93	87	68
Umbilical artery S/D	53	94	87	72
MCA PV	70	95	87	86
MCA PI	58	94	87	77
Fetal adrenal PI	25	78	40	64
Uterine vein p	80	87	87	90
Umbilical vein p	83	100	100	95
Ductus venosus p	71	86	62	90
Vein of galen p	53	100	100	68

## Discussion

**Figure 3:**
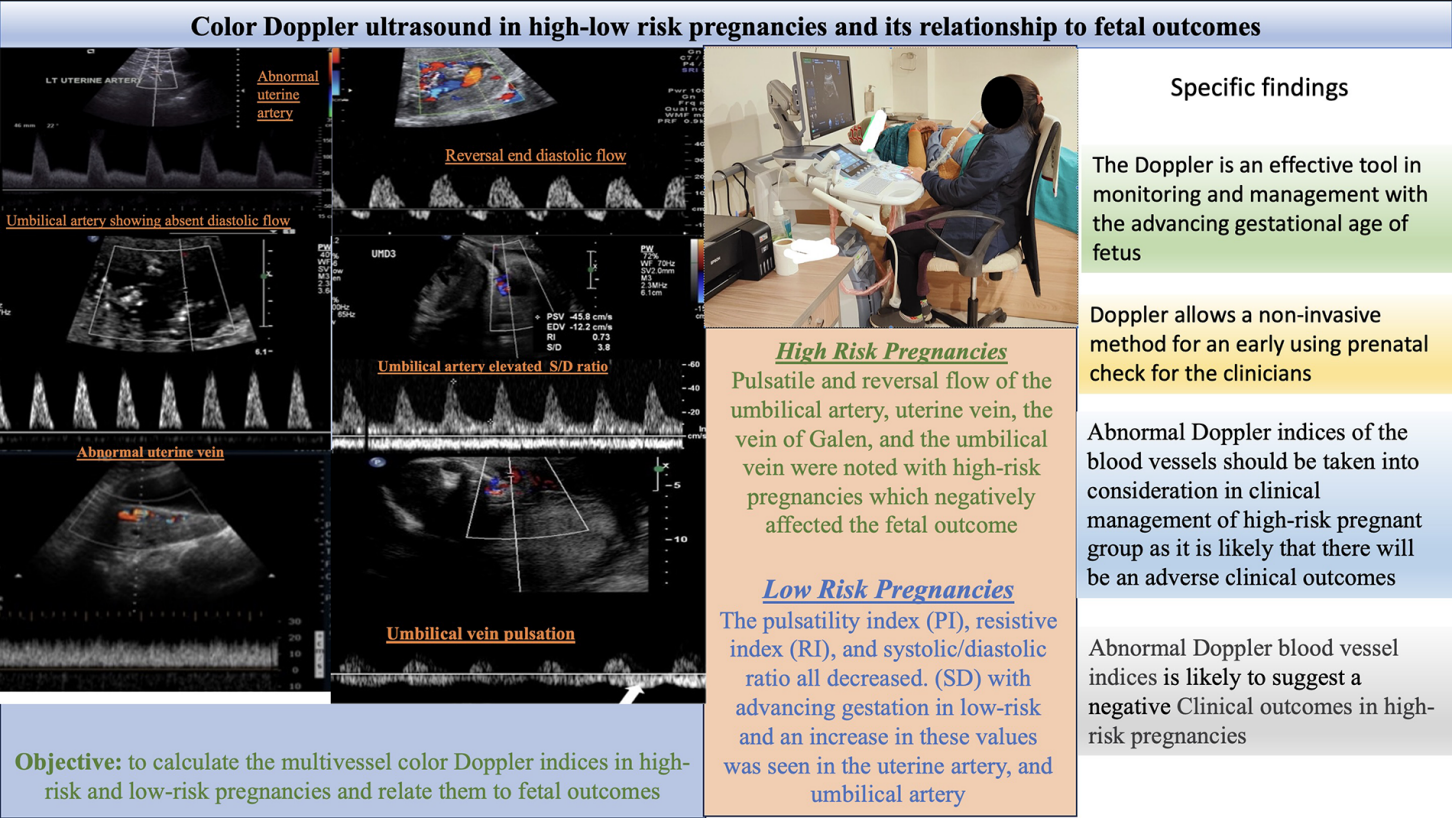
Color Doppler ultrasound in high-low risk pregnancies and its relationship to fetal outcome.

The present study used color Doppler ultrasound to examine the pregnancy outcomes of 60 participants displaying high- to low-risk factors. Color Doppler ultrasound, a non-invasive hemodynamic monitoring technology, is essential for maternal investigation of high-risk pregnancies. The technique can identify abnormal changes in the circulatory system such as increased vascular resistance and decreased diastolic flow (Figure 3) (4, 9). Studies have suggested that adopting Doppler ultrasound examination as a part of treatment has resulted in considerable declines in neonatal morbidity and mortality (7, 19, 20). Inaddition, Doppler ultrasound screening is helpful for high-risk pregnant women, but the benefits of the procedure have not been as well established for low- and unselected-risk pregnancies (21).

A maternal age of more than 35 years is a risk factor for fetal growth limitation. In our study, 53% of patients aged 30–34 were classified as at high risk. In contrast, 20–24 years (33%) was the dominant age group in the low-risk patients. Muhammad et al. reported limited fetal development in the younger age group (22), similar to our findings. In contrast to the low-risk group, where spontaneous birth was prevalent (80%), 83.3% of the high-risk group received cesarean sections, of which 30% were emergency procedures.

The mean RI, PI, and S/D ratios of the uterine artery between the two groups in the current study were noticeably different. As the gestation time increased, the values were reduced, while increasing in the high-risk group and declining in the low-risk group. Abnormal Doppler indices (70%–80%) were observed in the high-risk group; however, these variables had little to no effect on the perinatal outcome. Similar findings were obtained by Gomez et al. in their investigation of the sequential changes in the uterine artery PI over the first and second trimesters of pregnancy. They found that impedance decreased as gestation progressed. The outcomes of our study supported the hypothesis that complex pregnancies had greater PI and a higher prevalence of notching (23).

With the increased blood volume, the maternal venous system is as important as the arterial system. Changes in the venous pool govern heart function and sustain uteroplacental perfusion (24). In this work, we measured the flow velocity of the maternal uterine vein. Significant correlations were found between vein pulsations, birth weight, fetal distress, and newborn death in the high-risk group. Patients with pulsatile flow of the uterine wave have a greater occurrence of bilateral uterine artery notches, a risk factor in complicated pregnancies (25, 26). A study analyzing a chronic hypertensive population using the uterine artery identified high-risk pregnancies with poorer outcomes (27).

MCA-PSV has been reported to have a significant favorable connection with gestation (28). Fetal MCA accounts for about 7% of fetal cardiac output, indicating low-resistance circulation throughout pregnancy. The MCA is more sensitive to hypoxia and ischemia. The brain sparing that occurs in challenged fetuses increases diastolic flow and a drop in the pulsatility index. With increasing gestational age, we found that MCA PSV values significantly increased while PI values significantly decreased, consistent with past studies (29–32).

The current study showed that the velocity of the great cerebral vein, straight sinus, and transverse sinus increased with gestation. This may indicate that fetal cerebral blood flow increases with gestational age, followed by a decrease in diastolic central venous pressure that may be brought on by increased fetal heart wall compliance. Patients who experienced pulsations in their veins had a higher risk of fetal discomfort and death. The elevated central venous pressure accelerates the transmission of pressure waves to the periphery. Additionally, fetal cerebral venous arteries are more perfused and dilated due to the chronic hypoxia that permits the transmission of retrograde pressure waves from the heart (31).

In our study, 23% of the healthy fetuses had SS that was not pulsatile. This may have been because the turbulent blood flow at the confluence of the SS and SSS prevents the transmission of pressure waves from the fetal heart. Additionally, we discovered that the pulsations in the vein of Galen occurred 3.5 times more frequently than in the umbilical vein. The pulsating umbilical vein blood velocity spectrum is a more reliable predictor of worse neonatal outcomes than the GV (33).

The umbilical and portal circulations have constant, pulsating blood flow that is abnormal. However, a physiological pulsatile pattern occurs in the first trimester. Although rare, umbilical venous pulsations are believed to represent the final signs of fetal discomfort. According to Gudmundsson S. et al., irregular and diastolic umbilical cord venous pulses in fetuses with missing or reversed end diastolic flow in the umbilical artery may indicate a seriously injured fetus with a poor perinatal prognosis. These pulsations harm pregnancy, causing heart failure and severe fetal hypoxemia, with a high mortality rate (9, 25). In the current investigation, we observed that the low-risk group had normal flow; however, 13.3% and 6% of the patients in the high-risk group showed pulsations and reversed flow, respectively.

All microscopic artery channels and tertiary stem villi increase in quantity throughout a healthy pregnancy as the placenta grows. The umbilical artery's vascular resistance subsequently decreases as is normal. Due to diminished resistance vessel count in cases of umbilical placental insufficiency, the umbilical artery experiences increased resistance, thereby increasing the S/D, PI, and RI ratios (7, 34). Our study noted an increase in these values with advancing gestation. A typical pregnancy features a higher diastolic flow and lower indexes in the uterine and umbilical arteries. The diastolic flow in the uterine and umbilical arteries, in contrast, is reduced, missing, or even reversed in difficult pregnancies (7, 35, 36).

In the modern era, DV assessment is highly significant, as the course alters numerous fetal disorders such as cardiac function abnormality and fetal acidemia (24, 33). In our study, patients with abnormal PVIV displayed significantly reduced birth weight and increased fetal distress, factors that significantly increase neonatal mortality. In their investigation of diabetes pregnancies, Wong SF and colleagues discovered a similar association between ductus venosus abnormalities and poor fetal outcomes. This may be because fetal acidemia is related to increased DV indices (10, 13).

The fetal downstream aorta's normal blood flow is pulsatile and includes a low-end diastolic component. In the present study, PI readings decreased with increasing gestation, even though RI was unaltered. These outcomes corresponded to previous results (6). The hypothalamic-pituitary-adrenal axis is stimulated by intrauterine growth restriction and long-term fetal hypoxia, which causes the adrenal gland and associated vasculature to mature too rapidly. Tekay A. and Jouppila P. found that the PI values of the adrenal artery did not substantially alter with increasing gestation. In our study, there was no discernible difference in the adrenal artery blood flow measurements in either group (37).

Doppler measurements, particularly venous pulse, were found to be more sensitive to fetal prognosis for SGA. The specificity of the measurement, however, was comparatively lower. Regarding uterine waveform and venous pulsation, the venous and arterial Doppler tests demonstrated greater specificity. Delays in mediation in high-risk pregnancies due to worries about fetal issues may be the reason for the variation in sensitivity and specificity compared to a previous study (7). A study evaluating early-onset preeclampsia found that an uncomplicated pregnancy was distinguished by a lower venous pulse transit time in internal jugular veins, indicating higher venous vascular tone (38).

We found in the present study that Doppler indices were sensitive to outcomes in pregnancy. Moreover, in some research studies the eventual development of small-for-gestational age (SGA) neonates has been, significantly correlated with first-trimester uterine artery Doppler RI. When SGA is combined with pre-eclampsia, first-trimester uterine artery Doppler sensitivity is higher than when intrauterine growth restriction (IUGR) is used alone (39).

The women were only examined once throughout their pregnancy, which was the study's primary limitation and may have led to inconsistent results. Additionally, compared to low-risk pregnancies that typically resulted in spontaneous birth, high-risk pregnancies required more immediate intervention.

## Conclusion

Predicting unfavorable perinatal outcomes in high-risk obstetric patients using a non-invasive hemodynamic Doppler monitoring approach is now possible. Doppler imaging is more sensitive for early identification of fetal problems with subsequent perinatal outcomes compared to several other methods of prenatal evaluation. According to the present study, Doppler indices are essential for controlling high-risk pregnancies.

## Data Availability

The data supporting the conclusions of this article will be made available by the authors, without undue reservation, on request, adhering to the guidelines of the University.
